# Results of the Prolonged Use of Subcutaneous Continuous Infusion of Hydrocortisone in a Man with Congenital Adrenal Hyperplasia

**DOI:** 10.5402/2011/219494

**Published:** 2011-04-12

**Authors:** Emmanuel Sonnet, Nathalie Roudaut, Véronique Kerlan

**Affiliations:** Service d'Endocrinologie, Hôpital de la Cavale Blanche CHU de Brest, Boulevard Tanguy Prigent, 29609 Brest Cedex, France

## Abstract

This is a case report study of a young man with Congenital Adrenal Hyperplasia (CAH) who has been treated during 2 years by a subcutaneous continuous infusion hydrocortisone (SCIH) to optimize his treatment. Hydrocortisone was delivered via an insulin infusion device. We also studied the evolution of testicular adrenal rest tumors (TARTs) and the quality of life through SF36 survey. Four rates were determined, with a total of 47 mg per day. Biochemical parameters were normalized at 2 months. The SF36 questionnaire showed a progress of well-being. The weight decreased to 106 kg, that is, −5 kg (height: 1.71 m). Unfortunatly, there was no change of the TARTs. Two episodes of dermohypodermitis, with abscess at the infusion site, were observed. This case demonstrates the feasibility of prolonged SCIH therapy in patients with CAH, reporting positive effects on quality of life and on BMI.

## 1. Introduction

Congenital Adrenal Hyperplasia (CAH) due to 21-hydroxylase deficiency is a rare disease with glucocorticoid and mineralocorticoid deficiency. It is due to deletions or mutations of the cytochrome P450 21-hydroxylase gene (CYP21), which leads to an excess of ACTH secretion, adrenal hyperplasia with accumulation of steroid precursors such as 17 hydroxyprogesterone (17 OHP) [1]. The main goal of CAH assessment in adults is to replace deficient steroids in order to prevent adrenal crises and to suppress the abnormal secretion of androgens. In addition to fludrocortisone, different glucocorticoids can be used: prednisolone (BID), dexamethazone, even if the treatment of choice is hydrocortisone, thrice daily. Despite of this, the adequate replacement therapy is sometimes difficult to obtain.

Testicular Adrenal Rest Tumors (TARTs) are ACTH-dependent adrenal tissues contained in testis [2]. Due to their common embryologic origin, it is supposed that TARTs are derived from adrenocortical remnants descended with the testis. They are frequently present in CAH male adults [3] and seem to reflect undertreatment and to be associated with a reduced fertility [4]. A gain in fertility has been reported in this context through a suppressive glucocorticoid treatment [5].

Recent reports have shown that subcutaneous continuous infusion of hydrocortisone (SCIH) can be used in patients with adrenal insufficiency and CAH to replace endogenous cortisol production [6–8]. Our case report of the young CAH man, treated for 2 years, aimed to show the feasibility of such a prolonged therapy and its possible benefits, such as a reduction in TARTs volume.

## 2. Subject and Methods

### 2.1. Subject

A 27-year-old man with CAH, was followed up in our centre for 8 years. The disease was diagnosed at age of 2 weeks, after a salt-losing crisis. Diagnosis was confirmed later by molecular analysis of CYP21 gene showing 2 large deletions.

A therapy with fludrocortisone and hydrocortisone was started. After the development of puberty, the patient's treatment compliance decreased. Long periods with no therapy, and repeated hospitalizations for adrenal crisis were recorded. He was permanently tired. At the age of 22, testicular pain revealed the presence of bilateral TARTs. In this context, analyses showed azoospermia. Despite of our advice for 4 years, no conservation of sperm had been done. Volume of TARTs increased. Upon the ultrasound of the testis, the dimensions of the right testis's TARTs were 10 × 20 × 30 mm, and the left testis's TARTs showed a conglomerated, heterogeneous bulk of 49 × 21 × 42 mm.

A treatment with dexamethazone was introduced. But it induced an important weight gain and cutaneous atrophy. Due to patient's noncompliance, we observed major melanoderma. This noncompliance context, TARTs presence, and glucocorticoid therapy complications compelled us to deliver hydrocortisone by a continuous infusion using an insulin infusion device (Minimed 715, Medtronic Diabetes). Hemisuccinate of hydrocortisone was used (100 mg/mL). It was diluted to obtain 150 mg in 3 mL in line with 1UI on the pump delivering 0.5 mg of hydrocortisone. The basal infusion was set to normalize cortisol concentrations. Cortisol and 17 OHP were measured every 4 h during 24 h. Such patterns were performed before, at day 1, day 8, at 2 months, at year 1 and year 2 from the start of the therapy. Furthermore, fludrocortisone was delivered at 150 *μ*g per day, per os.

We also studied the evolution of TARTs through ultrasound, sperm count, and SF36 questionnaire's health score.

### 2.2. Assays

Serum total cortisol was measured by chemiluminescence (Vidas-Biomerieux). The intra-assay coefficient of variation (CV) was 3%, and the interassay CV was 5%. Results were in *μ*g/dl (conversion rate in nmol/L: 27.59).

Total testosterone, 17 OHP and Δ4 androstenedione (Δ4 AD) were measured with a radioimmunological assay (RIA) (Immunotech). Intra-assay CVs were, respectively, 2, 8, and 8.5%, interassay CVs 12, 9, and 12%. 

ACTH, plasmatic renin concentrations were measured by RIA (Cisbio Schering). Intra-assay CVs were, respectively, 6 and 9%, interassay CVs 10 and 5%.

## 3. Results

**Figure 1:**
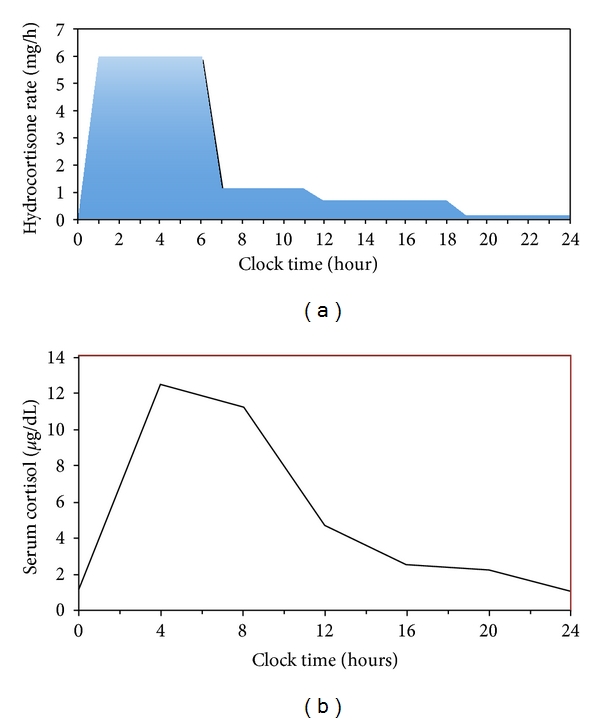
Hydrocortisone infusion rates used in the patient and serum cortisol concentration obtained after 2 months of hydrocortisone infusion.

Figure 1 shows the used basal infusion rates by the device. A total of 4 rates were set, ranging from 0.1–1.6 mg/h. A total of 47 mg per day was delivered (0.42 mg/kg/day). The rates were unchanged after 2 months of infusion.  Table 1 presents the evolution of different biochemical parameters.

Shortly after the initiation of the hydrocortisone infusion, the patient felt less tired. He was more dynamic, and made important decisions concerning his professional and private life (found a stable job, obtained his driving licence, established a steady and romantic relationship). The SF36 questionnaire, realized before the use of the device, and 1 year after, showed an increase of the well-being (physical function: from 70 to 95). The weight decreased to 106 kg (−5 kg, 1.71 m; BMI: 36.25 kg/m^2^). 

Unfortunatly, despite of the good biological results, there was no decrease in the TARTs' volume (the dimensions remained stable). The azoospermia persisted. Two episodes of dermohypodermitis, with abscess at the infusion site, were observed at years 1 and 2 of the start of the infusion. We reported no episode of illness or big stress imposing to double the rates. No hospitalization was necessary due to a possible adrenal crisis.

## 4. Discussion

In this work, we report the case of a male patient with CAH who was treated by prolonged subcutaneous infusion of hydrocortisone. The cortisol circadian delivery rhythm was obtained quickly, as well as satisfying biological results, that is, a good CAH control and an increased well-being. Unfortunatly, after 2 years of SCIH, there was no decrease of the TARTs volume. Two episodes of dermohypodermitis were recorded.

To our knowledge, this is the first case report evaluating prolonged SCIH therapy in an adult with CAH. Prior published studies have shown the interest of a circadian delivery of hydrocortisone in 2 patients with Addison's disease, 2 patients with CAH [7], and the technical feasibility of this hydrocortisone infusion in 7 patients with Addison's disease during 12 weeks [6]. More recently, Bryan et al. reported the case of a young boy treated during 4 years by SCIH [8]. His therapy made possible a normal progression of puberty. In these described CAH patients with SCIH, the hydrocortisone was delivered with an insulin pump, set at 4 or 5 different rates. The course of the basal rates was not very different from one patient to another. The maximal reported rate was 3 mg/h. But for our patient, the maximal one was higher (6 mg/h), as well as the total daily dose. This higher dose could be due to a different indication, an older age or a higher weight. These basal rates were set to be near the normal secretion rates, as reported in other studies [9].

The use of SCIH induced a fast decrease of the 17 OHP and ACTH levels, as described earlier [7, 8]. These optimal results persisted for 2 years. In our patient, Δ4 AD levels, decreased at first, but remained high despite of the normalized ACTH concentrations. This was not reported by Bryan et al. [8]. Furthermore, we found appropriate levels of total testosterone, despite of the presence of TARTs. Unfortunatly, we did not measure the progesterone (which could have influenced the testosterone production) and FSH, and did not study the possible conversion of adrenal androgens through dexamethazone testing.

This therapy was very well tolerated. The patient managed the pump himself. But we noticed two episodes of dermohypodermitis, with a local abscess at the infusion site. Similar episodes were reported by Bryan et al., but only one in 4 years [8]. Local abscess is very rare with infusion of insulin. It is difficult to know if the infusion of hydrocortisone could enhance such a local complication. The method to change patient's catheter with a perfect skin preparation was revised regularly. But it did not prevent the apparition of this skin complication.

TARTs are frequent in young male CAH patients [10]. It seems they could even appear in subjects with appropriate treatment compliance but are more frequent in males with chronic undertreatment [4]. Claahsen-van der Grinten et al. demonstrated reversing of TARTs, in a man, through a suppressive glucocorticoid treatment [5]. So we hypothesized that a good hormonal control during 2 years could induce a decrease in the TARTs therefore reversing the infertility. Unfortunatly, TARTs remained stable in time. Different hypotheses could explain this: (a) the intensification of glucocorticoid treatment may not be always sufficient in reducing the TARTs, as suggested in a study with the hypothesis of an abnormal control of testis development, independent of the ACTH stimulation [11]; (b) the duration of the SCIH could be too short; (c) the lesions in the testicular glands of our patient could be irreversible [11]. To confirm this last hypothesis, we suggest an earlier application of SCIH in young males with TARTs responsible of a compression of rete testis and oligospermia due to obstruction of seminiferous tubules, but without fibrosis.

The use of SCIH seems to have other positive effects. Despite of therapy's constraints, we noticed an increase in our patient's well-being through the prospectively observed progression of SF36 health score. Upon morning rise, he was dynamic and stayed active in his daily tasks. A decreased quality of life in patients with Addison's disease or CAH is well known [12]. The influence of the mode of the hydrocortisone replacement has been noted [13]. Thus, SCIH therapy capable of mimicking the natural circadian cortisol production seems to be the best hydrocortisone delivery mode enhancing quality of life. Furthermore, we reported patient's weight loss to be beneficial for most CAH adults having high BMIs [14]. This reduction of BMI was also described by Bryan et al. [8].

SCIH represents a new approach to treat CAH. Even though we did not analyze this treatment mode's cost, we would like to emphasize that through a gain in health score and patient's well-being, less frequent hospitalization will be required. This can lead to significant savings in national public health's budget.

In conclusion, this case demonstrates the feasibility of prolonged SCIH therapy in patients with CAH. This method could have positive effects on quality of life and on BMI. Unfortunatly, we did not achieve a decrease of the TARTs. This method is a new and promising method, with the aim to control the biochemical parameters, and to diminish complications of CAH and glucocorticoid treatment. Further studies are needed to better identify the role and benefits of this new delivery mode, through a larger selection of patients and comparing this new therapy's outcomes with those of conventional therapy.

## Figures and Tables

**Table 1:** Effect of hydrocortisone infusion on biochemical parameters, before, 2 months and 2 years of hydrocortisone infusion (all blood samples were taken at 8 h; 17 OHP: 17 hydroxyprogesterone, Δ4 AD: delta 4 androstenedione).

	Before	2 months	2 years
Cortisol (*μ*g/dl) (*N*: 12–25)	1.5	11.2	13.7
17 OHP (ng/mL) (*N*: <2)	86	24.5	26.5
ACTH (pmol/L) (*N*: 2–12)	494	15.5	26
Δ4 AD (ng/mL) (*N*: <1.8)	24.6	10.8	16.5
Testosterone (ng/mL) (*N*: 3–10)	11.8	3.3	7.6
